# Exosomal protein angiopoietin-like 4 mediated radioresistance of lung cancer by inhibiting ferroptosis under hypoxic microenvironment

**DOI:** 10.1038/s41416-022-01956-7

**Published:** 2022-09-01

**Authors:** Yuhong Zhang, Xinglong Liu, Liang Zeng, Xinrui Zhao, Qianping Chen, Yan Pan, Yang Bai, Chunlin Shao, Jianghong Zhang

**Affiliations:** Institute of Radiation Medicine, Shanghai Medical College, Fudan University, 200032 Shanghai, China

**Keywords:** Tumour-suppressor proteins, Predictive markers, Molecular medicine

## Abstract

**Background:**

Hypoxia-mediated radioresistance is a major reason for the adverse radiotherapy outcome of non-small cell lung cancer (NSCLC) in clinical, but the underlying molecular mechanisms are still obscure.

**Methods:**

Cellular and exosomal ANGPTL4 proteins under different oxygen status were examined. Colony survival, lipid peroxidation and hallmark proteins were employed to determine the correlation between ferroptosis and radioresistance. Gene regulations, western blot and xenograft models were used to explore the underlying mechanisms of the role of ANGPTL4 in radioresistance.

**Results:**

ANGPTL4 had a much higher level in hypoxic NSCLC cells compared to normoxic cells. Up- or down- regulation of ANGPTL4 positively interrelated to the radioresistance of NSCLC cells and xenograft tumours. GPX4-elicited ferroptosis suppression and lipid peroxidation decrease were authenticated to be involved in the hypoxia-induced radioresistance. ANGPTL4 encapsulated in the exosomes from hypoxic cells was absorbed by neighbouring normoxic cells, resulting in radioresistance of these bystander cells in a GPX4-dependent manner, which was diminished when ANGPTL4 was downregulated in the donor exosomes.

**Conclusion:**

Hypoxia-induced ANGPTL4 rendered radioresistance of NSCLC through at least two parallel pathways of intracellular ANGPTL4 and exosomal ANGPTL4, suggesting that ANGPTL4 might applicable as a therapeutic target to improve the therapeutic efficacy of NSCLC.

## Introduction

Lung cancer, as a malignant tumour originating from abnormal bronchial epithelial tissue, is the main cause of cancer-related death worldwide. Lung cancers are classified into small cell lung cancer (SCLC) and non-small cell lung cancer (NSCLC) according to their morphological characteristics and biological behaviours. Among them, about 85% of patients are NSCLC [1]. At present, the treatment of NSCLC mainly adopts surgery, drug therapy (including systemic chemotherapy, targeted therapy and immunotherapy), radiotherapy and local intervention. Radiotherapy can be used alone or in combination with other treatments and thus is an important mean in lung cancer treatment [2]. However, there are still some patients with a poor prognosis due to tumour radioresistance so that their 5-year survival rate is less than 17% [3]. Although the mechanisms of radioresistance have been extensively explored in terms of genes, signalling pathways and interactions with immune cells, the underlying issues remain unclear [4, 5]. Investigation of these problems will provide strong support for improving the clinical benefits of current anticancer therapy strategies.

Tumour microenvironment (TME) is a unique environmental network composing of a variety of non-malignant cells, tumour cells, extracellular matrix (ECM), surrounding blood vessels, and cell secreted products such as exosomes, cytokines and chemokines [6]. Hypoxia, a prominent feature of TME, has long been considered as a critical regulator of radioresistance. It can alter the expression of pro-survival genes and then mediate cell–cell interaction through extracellular environmental factors [7, 8]. Recently, the roles of exosomes in tumorigenesis and lung cancer development have attracted more and more attention [9]. Exosomes are defined as a class of extracellular vehicles (EVs) formed by inward budding of endosomal membrane and releasing into extracellular TME upon fusion with the plasma membrane [10, 11]. Importantly, within hypoxic TME, the tumour-derived exosomes (TDEs) are delivering tumoral or hypoxic signalling to both surrounding tumour cells and stromal cells, and thus play indispensable functions in the TDE-mediated tumour cell communications. With the deepening of relevant researches [12], an idea has been speculated that the crosstalk of hypoxia, exosomes and TME biomolecules may provide novel insights into tumour radioresistance.

Recently, the role of ferroptosis in tumour radiosensitivity has become a very attractive topic [13, 14]. Ferroptosis, an iron-dependent form of cell death, is characterised by the loss of lipid peroxide repair through glutathione peroxidase 4 (GPX4). The increases of free iron and the oxidation of polyunsaturated fatty acid-containing (PUFA-containing) phospholipids have emerged as a potent mechanism for tumour development and radiation response [15]. GPX4 can reduce glutathione and convert lipid hydroperoxides to lipid alcohols, thereby mitigates lipid peroxidation and inhibits ferroptosis [16]. The cystine/glutamate antiporter SLC7A11, as a multi-pass transmembrane protein mediating ferroptosis, assists cells to obtain cysteine by modulating the activity of cystine/glutamate antiporter [17]. Previous studies have identified that radiation can inactivate SLC7A11 and GPX4, thereby accumulate lipid peroxidation and trigger ferroptosis [18, 19]. Nonetheless, there is a lack of evidence to directly demonstrate the relationship between ferroptosis and tumour radioresistance, especially underlying crosstalk of TME components under hypoxia.

Moreover, as a secretory glycoprotein, ANGPTL4 protein is widely regarded as a decisive regulator of angiogenesis and an inflammatory carcinogenic mediator that participates in the occurrence of human cancer in the manner of autocrine and paracrine activity [20–22]. ANGPTL4 is extensively expressed in liver, vascular system, hematopoietic system and other tissues, and participates in physiological and pathological processes through different molecular mechanisms. Kim et al. found that prostaglandin E_2_ (PGE_2_) played an important role in promoting colorectal cell proliferation through ANGPTL4 under hypoxic condition [23]. A number of studies have shown that *ANGPTL4* is an important ferroptosis-related gene [24]. As a direct transcriptional co-activator of PDZ-binding motif (TAZ)-regulated target gene, *ANGPTL4* sensitised ferroptosis through activation of NADPH Oxidase 2 (NOX2). Given that ANGPTL4 has emerged as a crucial player in cancer progression, it is important to know whether ANGPTL4 undergoes the regulation of tumour radioresistance under hypoxia and how its contribution in the signalling communication within TME.

In this study, we found that the extracellular ANGPTL4 protein was enriched in hypoxic tumour-derived exosomes and endowed radioresistance of NSCLC cells under hypoxia. When ANGPTL4 was absorbed by normoxic NSCLC cells through exosomes transport, the radiosensitivity of these normoxic cells was also reduced. Importantly, inhibition of ferroptosis mediated by extracellular ANGPTL4 as well as hypoxic exosomal ANGPTL4 also promoted radioresistance in NSCLC cells. How ANGPTL4 to mediate the association between ferroptosis and tumour radioresistance under hypoxic microenvironment was further investigated.

## Materials and methods

The materials and methods can be found in the Supplementary Materials and Methods.

## Results

### High expression and secretion of ANGPTL4 in hypoxic lung cancer cells

**Fig. 1:**
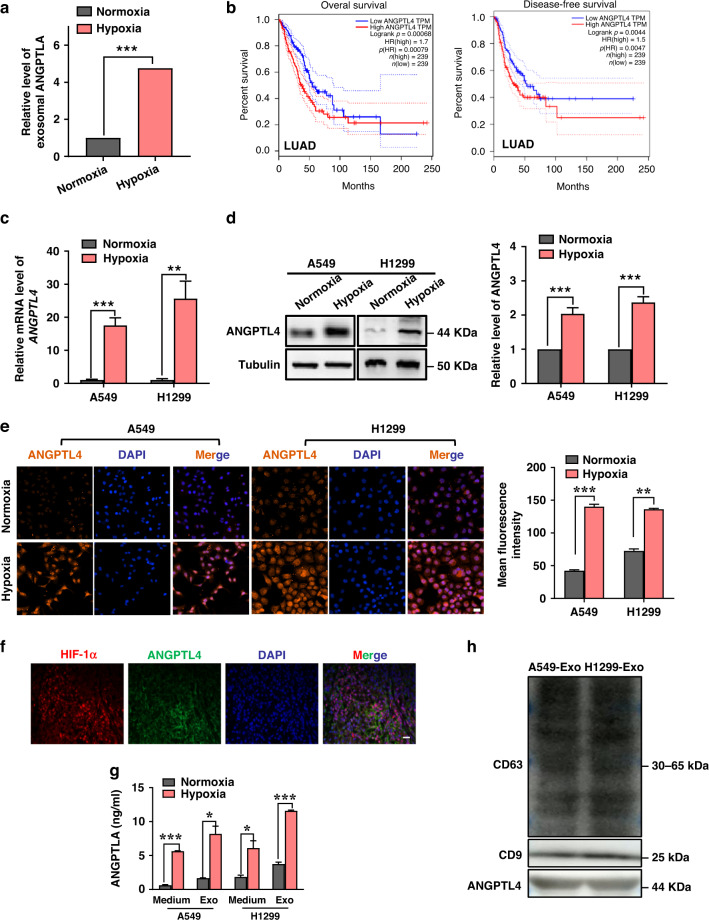
Hypoxia induces intracellular expression and extracellular secretion of ANGPTL4. **a** Relative exosomal ANGPTL4 levels in normoxic and hypoxic A549 cells measured by tandem mass tag (TMT) quantitative proteomics. **b** High expression of ANGPTL4 predicted a poor prognosis in lung adenocarcinoma based on the Gene Expression Profiling Interactive Analysis (GEPIA) database. **c**, **d** qRT-PCR and western blot analyses of ANGPTL4 expressions in A549 and H1299 cells at 48 h after hypoxia treatment in comparison with normoxia. **e** Representative immunofluorescence images and quantification of the expression of ANGPTL4 in A549 and H1299 cells under hypoxic or normoxic conditions. Cell nuclei were labelled with DAPI dihydrochloride. Scale bar, 20 μm. **f** ANGPTL4 was predominantly presented in HIF-1α-expressing hypoxic regions of A549 xenograft tumours (×400). Scale bar, 20 μm. **g** ELISA assay of ANGPTL4 protein in the cell culture medium and exosomes derived from the A549 and H1299 cells under hypoxia or normoxia. **h** Western blot analyses of CD63, CD9 and ANGPTL4 expressions in the exosomes from A549 and H1299 cells. *P < 0.05; **P < 0.01; ***P < 0.001.

ANGPTL4 is inextricably linked with angiogenesis and also participates in various steps of inflammation and tumour development through autocrine and paracrine forms [25]. Our previous study using tandem mass tag (TMT) quantitative proteomic analysis [26] has demonstrated that ANGPTL4 encapsulated in hypoxic lung cancer cell-derived exosome cargoes was richer than that in normoxic exosomes. Their relative amount was illustrated in Fig. 1a. Using Gene Expression Profile Interaction Analysis (GEPIA), we found that upregulation of ANGPTL4 predicted the poor prognosis in patients of lung adenocarcinoma (LUAD) (Fig. 1b) as well as adrenocortical carcinoma (ACC), stomach adenocarcinoma (STAD) and brain lower grade glioma (LGG) (Supplementary Fig. s1), suggesting that ANGPTL4 has a potential to be a therapeutic target. Compared with normoxia, hypoxia treatment resulted in robust induction of ANGPTL4 in human lung cancer A549 and H1299 cells (Fig. 1c–e). In situ immunofluorescence assay showed that ANGPTL4 was expressed synregionally with HIF-1α at high levels in mouse xenograft tumours (Fig. 1f). Since HIF-1α is the gold standard for identifying hypoxia, this result suggests that ANGPTL4 may also be a conspicuous molecule distinguishing hypoxic tumour cells from normoxic cells.

Extracellular vesicles, including exosomes, are important components of tumour hypoxic microenvironment, which in turn affect the type and quantities of proteins carried by exosomes [27]. According to the mature exosome extraction method, we have characterised the exosomes isolated from NSCLC cells by TEM, nanoparticle tracking analysis and western blotting assay [26]. Figure 1g illustrates that ANGPTL4 was concentrated in both supernatant and exosomes of A549 and H1299 cells and it had a much higher level under hypoxia than that under normoxia. Notably, the content of ANGPTL4 in exosomes was significantly higher than that of its corresponding supernatant, suggesting that ANGPTL4 was mainly packed in exosomes and corresponded to the change of intracellular ANGPTL4 expression. Meanwhile, we also confirmed that ANGPTL4 was enriched in exosomes by the specific exosomal markers, CD63 and CD9 (Fig. 1h).

### ANGPTL4-modulated radioresistance under hypoxia

**Fig. 2:**
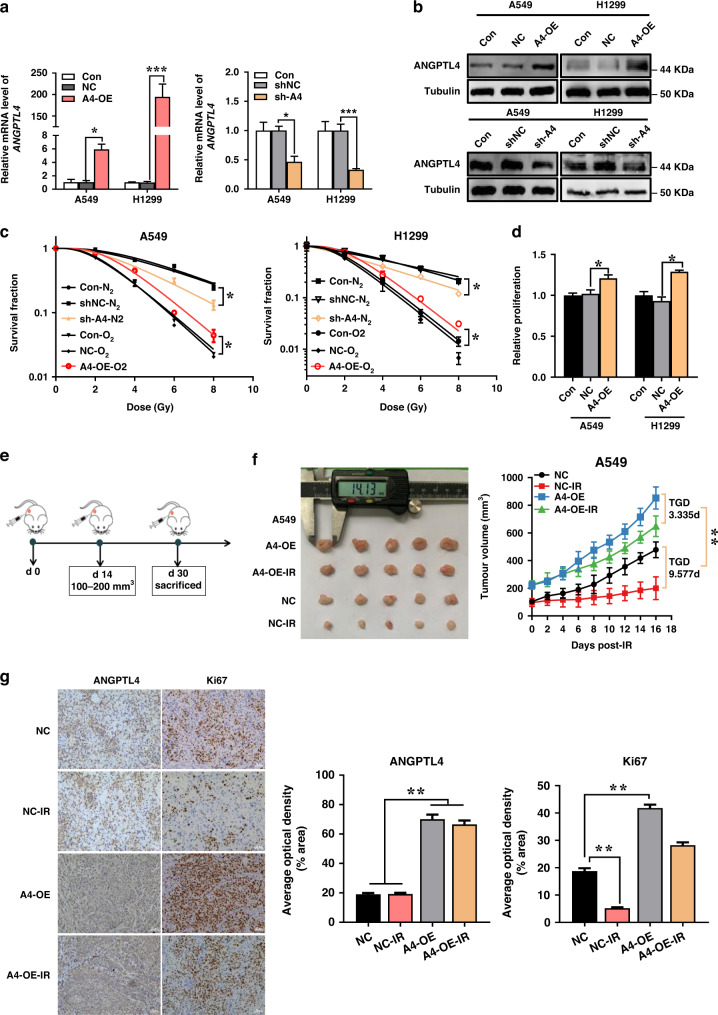
Upregulation of ANGPTL4 augments hypoxia-induced radioresistance of NSCLC in vivo and in vitro. **a**, **b** qRT-PCR and Western blot analyses of *ANGPTL4* expression in A549 and H1299 cells stably transfected with sh-ANGPTL4, ANGPTL4-OE or their negative control shNC and ANGPTL4-OE-NC, respectively. A4 and OE are the abbreviations of ANGPTL4 and overexpression, respectively. **c** Clonogenic survivals of A549 and H1299 cells with or without ANGPTL4 overexpression under normoxia and sh-ANGPTL4 transfection under hypoxia. **d** Quantification of the proliferation of A549 and H1299 cells transfected with ANGPTL4-OE or ANGPTL4-OE-NC. **e** The schematic diagram of the in vivo experimental design for xenograft establishment and irradiation. **f** Representative xenograft tumour mass (*n* = 5) and the tumour growth curves of A549 cells transfected with ANGPTL4-OE or ANGPTL4-OE-NC with or without local irradiation of X-rays at a single dose of 20 Gy. **g** Representative images and quantification of immunohistochemical staining of Ki67 and ANGPTL4 in xenograft tumours with indicated treatments (*n* = 5). Scale bars, 50 μm. *P < 0.05; **P < 0.01.

To further explore the function of ANGPTL4 in hypoxic tumour radioresistance, the cells stably transfected with lentivirus-mediated ANGPTL4 interference (sh-ANGPTL4) and overexpression (ANGPTL4-OE) were constructed and verified by qRT-PCR and Western blot assay, respectively (Fig. 2a, b and Supplementary Fig. s2). As expected, the radiosensitivities of A549 and H1299 cells under hypoxia were significantly lower than those under hypoxic condition (Fig. 2c). However, the radioresistance under hypoxic condition was significantly reversed by sh-ANGPTL4 transfection. Conversely, ANGPTL4 overexpression enhanced radioresistance even in normoxic cells. Therefore, a high level of ANGPTL4 could significantly increase the resistance of NSCLC cells to irradiation.

Notably, ANGPTL4 overexpression significantly enhanced the proliferation of A549 and H1299 cells (Fig. 2d). Next, we subcutaneously inoculated A549 cells transfected with ANGPTL4-OE into the left flanks of male nude mice to construct a xenograft model (Fig. 2e). When the tumours approached to 100–200 mm^3^ on the 14th day after tumour cell seeding, they were locally irradiated with X-rays at a single dose of 20 Gy and the tumour volumes were measured every other day. Consistent with the results of in vitro experiments, ANGPTL4 overexpression facilitated the tumour growth and reduced the anti-tumour effect of radiotherapy (Fig. 2f). TGD of NC-IR and A4-OE-IR group was 9.58 d and 3.35 d, respectively. Moreover, the immunohistochemistry assay confirmed that ANGPTL4 possessed a higher average optical density (AOD) in A549-ANGPTL4-OE xenograft tumour compared to its NC group, and irradiation reduced the AOD level of Ki67 i.e. inhibited cell proliferation (Fig. 2g). But the overexpression of ANGPTL4 significantly increased Ki67 expression level in tumour tissue, indicating again that ANGPTL4 protected cells from irradiation and promoted cell proliferation.

### Hypoxia promoted radioresistance through inhibition of ferroptosis

**Fig. 3:**
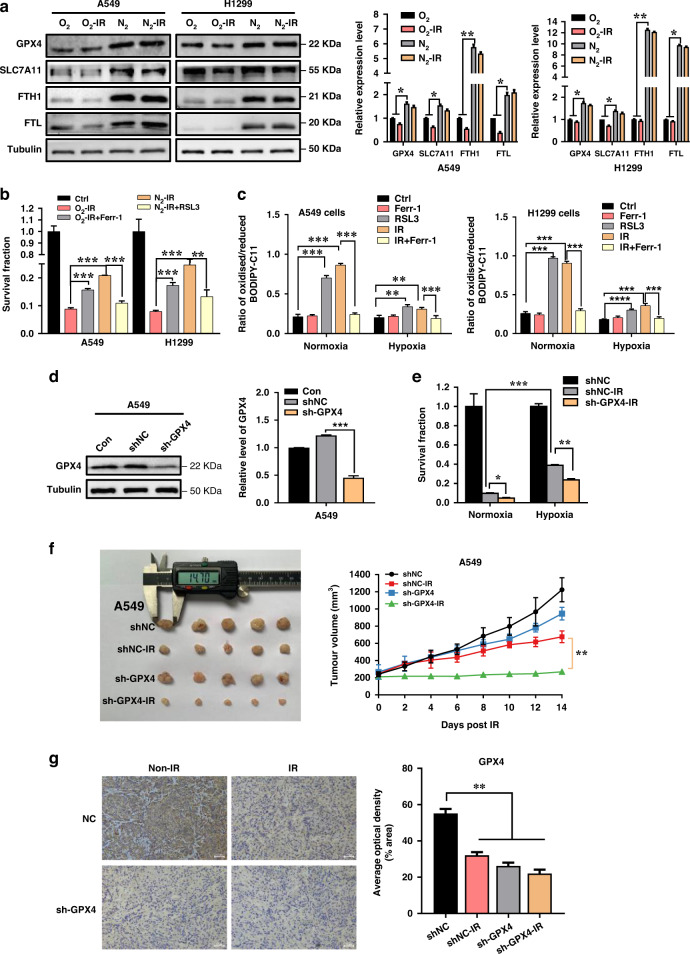
Ferroptosis is involved in hypoxia-induced radioresistance. **a** Western blot assay of GPX4, SLC7A11, FTH1, FTL proteins and their relative expression levels in A549 and H1299 cells. **b** Clonogenic survivals of A549 and H1299 cells that were respectively pretreated with 5 μM ferrostatin-1 (Ferr-1) under normoxia, 5 μM RSL3 under hypoxia, or 0.1% DMSO as drug control for 24 h followed by 6 Gy γ-ray irradiation. **c** The lipid peroxidation levels (represented by the ratio of oxidised/reduced C11 BODIPY) in normoxic and hypoxic A549 and H1299 cells pretreated with Ferr-1 and RSL3 for 24 h prior to 6 Gy γ-ray irradiation. **d** Western blot analysis and quantification of GPX4 expression level in A549 cells stably transfected with sh-GPX4 or it shNC. **e** Clonogenic survivals of normoxic and hypoxic A549 cells stably transfected with sh-GPX4 or its shNC followed by 6 Gy γ-ray irradiation. **f** Representative xenograft tumour mass (*n* = 5) and the tumour growth curves of A549 cells transfected with sh-GPX4 or shNC with or without local irradiation of X-rays at a single dose of 20 Gy. **g** Representative images and quantification of immunohistochemical GPX4 staining in tumour tissues of above-indicated groups. Scale bars, 50 μm. *P < 0.05; **P < 0.01; ***P < 0.001.

In response to hypoxia, the survival pathways of cancer cells can be activated, leading to the occurrence of radioresistance. Given that ferroptosis could be regulated by hypoxia, we hypothesised that ferroptosis played a key role in radioresistance under hypoxia, and thus we detected the expressions of ferroptosis inhibition-related proteins of GPX4, SLC7A11, ferritin heavy chain (FTH1) and ferritin light chain (FTL) in A549 and H1299 cells exposed to γ-rays under normoxic and hypoxic conditions. It was found that the expressions of these proteins were reduced to different levels by irradiation but all increased in response to hypoxia compared to normoxia (Fig. 3a), indicating that irradiation triggered ferroptosis but hypoxia treatment suppressed ferroptosis in NSCLC cells. To further substantiate this observation, we treated normoxic cells and hypoxic cells with ferroptosis inhibitor Ferr-1 and ferroptosis inducers RSL3, respectively, prior to 6 Gy irradiation and found that Ferr-1 increased the survival of irradiated normoxic cells and RSL3 reduced the survival of irradiated hypoxic cells (Fig. 3b). Moreover, we detected the accumulation of lipid peroxidation (a hallmark of ferroptosis) in NSCLC cells. Under the normoxic condition, both irradiation and RSL3 treatment enhanced the lipid peroxidation level in NSCLC cells, but this radiation response was eliminated by ferroptosis inhibitor Ferr-1 (Fig. 3c and Supplementary Fig. s3). Under hypoxia, irradiation and RSL3 treatment also increased the abnormal lipid peroxidation in NSCLC cells, but the degree was lower than that of normoxic cells.

Next, we transfected A549 cells with GPX4 shRNA (sh-GPX4) to establish a stable GPX4-knockdown cell line (Fig. 3d) and found that sh-GPX4 aggravated irradiation-induced cell death under both normoxic and hypoxic conditions (Fig. 3e). When A549 cells transfected with shNC or sh-GPX4 were subcutaneously injected into athymic nude mice, single dose irradiation of 20 Gy significantly inhibited the growth of xenografts, which was much effective due to GPX4 knockdown (Fig. 3f). TGDs of shNC-IR and sh-GPX4-IR groups were 6.34 d and 13.73 d, respectively. Moreover, the expression of GPX4 in the tumour tissue was confirmed to be downregulated by sh-GPX4 transfection and significantly reduced by tumour irradiation (Fig. 3g). Therefore, ferroptosis had a negative correlation with radioresistance.

### Relationship of ANGPTL4 and ferroptosis

**Fig. 4:**
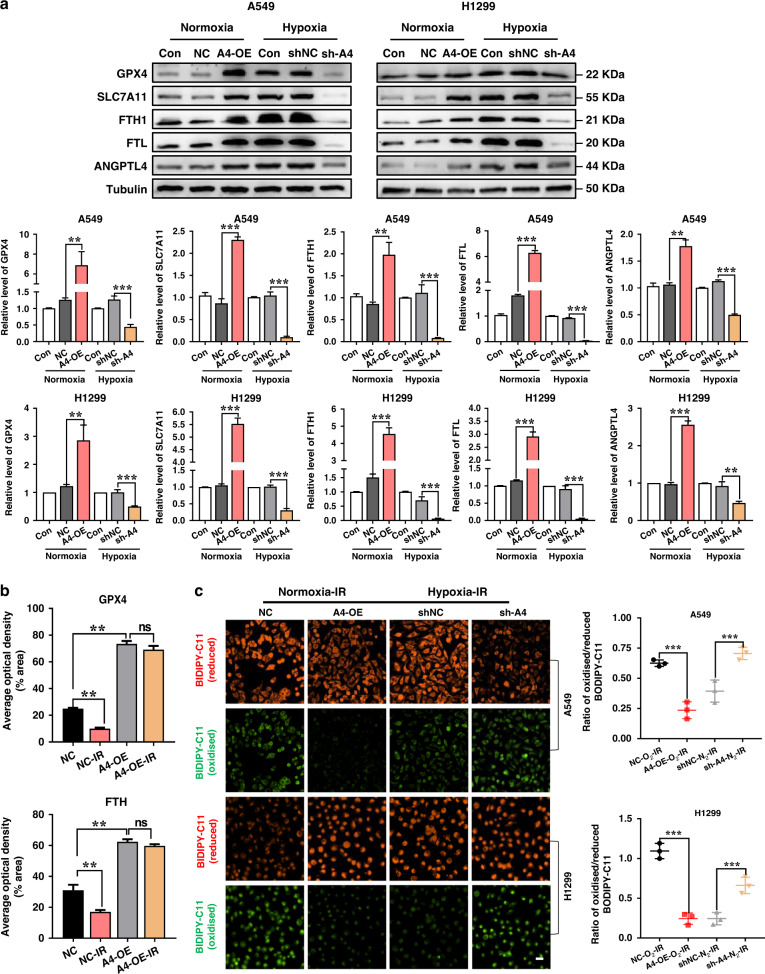
Hypoxia-induced ANGPTL4 contributes to radioresistance by inhibiting ferroptosis in NSCLC cells. **a** Western blot assay of GPX4, SLC7A11, FTH1, FTL proteins and their relative expression levels in normoxic A549 and H1299 cells stably transfected with ANGPTL4-OE or its negative control (NC), and in hypoxic cells stably transfected with sh-ANGPTL4 or shNC. **b** Quantification of the average optical densities of FTH1 and GPX4 staining in the tissues of xenograft tumour generated from A549 cells transfected with ANGPTL4-OE or its NC (*n* = 5). Tumours of IR group were irradiated with X-rays at a single dose of 20 Gy. **c** Representative immunofluorescence images and quantification of lipid peroxidation in normoxic A549 and H1299 cells with ANGPTL4 overexpression (A4-OE) and hypoxic A549 and H1299 cells with ANGPTL4 interference (sh-A4). Cells in IR group were irradiated with 6 Gy γ-rays. Scale bar, 20 μm. **P < 0.01; ***P < 0.001.

To know the relationship of ANGPTL4 and ferroptosis, we checked the expressions of ferroptosis hallmark proteins in the ANGPTL4-modulated NSCLC cells under normoxic and hypoxic conditions. It was found that the expressions of GPX4, SLC7A11, FTH1 and FTL proteins were all increased in ANGPTL4-overexpressed cells under normoxia and decreased in ANGPTL4-knockdown cells under hypoxia (Fig. 4a). This relationship was also confirmed in vivo. It was found that the expressions of GPX4 and FTH1 in A549 xenograft tumours were significantly reduced by irradiation, which was reversed by the upregulation of ANGPTL4 (Fig. 4b and Supplementary Fig. s4). On the other hand, irradiation-induced lipid peroxidation was drastically reduced in normoxic NSCLC cells due to the upregulation of ANGPTL4, and it was significantly increased in hypoxic NSCLC cells due to the downregulation of ANGPTL4 (Fig. 4c). In addition, treatment of ANGPTL4-knockdown cells with ferroptosis inhibitor of Ferr-1 restored hypoxia-induced radioresistance (Supplementary Fig. s5). Conclusively, these results suggested that ANGPTL4 had a radiation protective effect by reducing the accumulation of lipid peroxidation and restraining ferroptosis.

### ANGPTL4 was essential for the hypoxic exosomes-induced radioresistance to bystander NSCLC cells

**Fig. 5:**
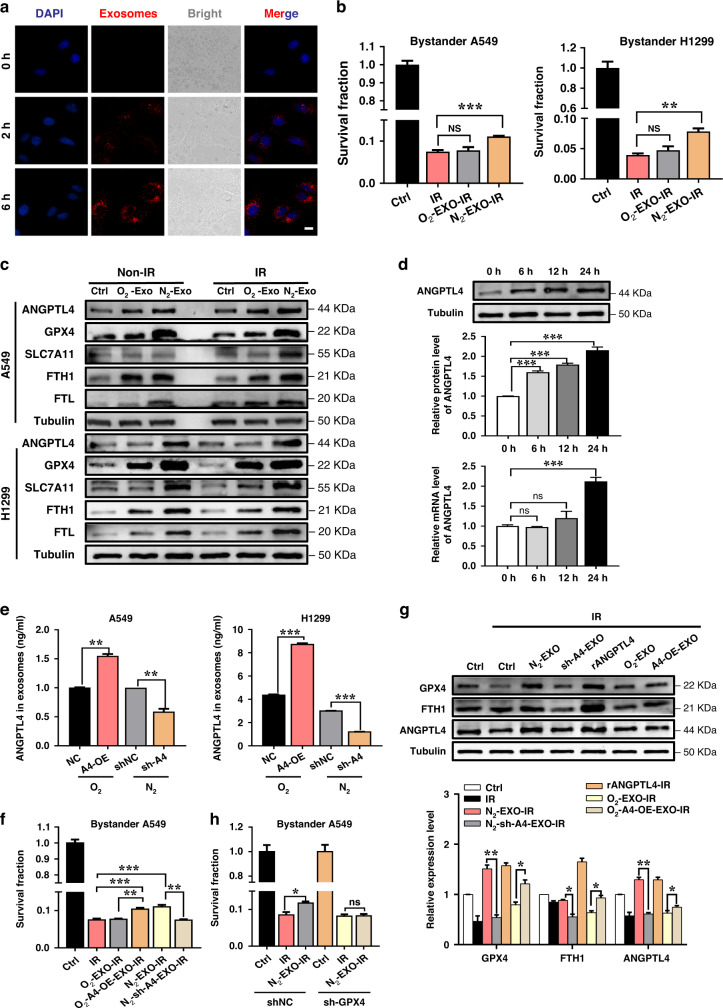
ANGPTL4 is essential for hypoxic exosome-induced radioresistance of bystander cells. **a** Representative fluorescence images of exosomes uptake into H1299 cells in a time-dependent manner. Scale bar, 10 μm. **b** Clonogenic survivals of A549 and H1299 cells that were co-cultured with homologous N_2_-EXO or O_2_-EXO for 24 h followed by 6 Gy γ-ray irradiation. **c** Western blot assay of GPX4, SLC7A11, FTH1, FTL, ANGPTL4 proteins and their relative expression levels in A549 and H1299 cells that were co-cultured with its homologous O_2_-EXO and N_2_-EXO with or without γ-ray irradiation. **d** The transcription and protein levels of ANGPTL4 in bystander A549 cells after co-culturing with its exogenous N_2_-EXO for indicated times. **e** Relative level of ANGPTL4 protein in exosomes derived from normoxic A549 and H1299 cells transfected with ANGPTL4-OE or from hypoxic A549 and H1299 cells transfected with sh-ANGPTL4. **f** Clonogenic survivals of A549 cells that were co-cultured with homologous N_2_-EXO, N_2_-sh-A4-EXO, O_2_-EXO, O_2_-A4-OE-EXO for 24 h prior to 6 Gy γ-ray irradiation. **g** Western blot assay of GPX4, FTH1, ANGPTL4 proteins and their relative expression levels in A549 cells that were co-cultured with it homologous N_2_-EXO, N_2_-sh-A4-EXO, O_2_-EXO, O_2_-A4-OE-EXO or treated with human recombinant ANGPTL4 protein (rANGPTL4) followed by 6 Gy γ-ray irradiation. **h** Clonogenic survivals of sh-GPX4 transfected A549 cells that were co-cultured with homologous N_2_-EXO followed by 6 Gy γ-ray irradiation. *P < 0.05; **P < 0.01; ***P < 0.001.

Tumour radiosensitivity is always influenced by its environment containing exosomes [28]. To know whether the exosomes released from tumour cells affect the radiation response of bystander cells, we co-cultured normoxic H1299 cells with its exosomes labelled with PKH26 and found that these exosomes were endocytosed into the recipient cells within 2 h, and this uptake continually increased with culture time (Fig. 5a). When normoxic NSCLC cells were cultured together with 10 μg homologous exosomes from normoxic or hypoxic cells for 24 h, their survival after 6 Gy irradiation were significantly increased by the hypoxic exosomes (N_2_-EXO) but just slightly increased (*P* > 0.05) by the normoxic exosomes (O_2_-EXO) (Fig. 5b). To consolidate the effect of hypoxic exosomes (N_2_-EXO) on the radiation response of bystander cells, we further studied whether inhibition of exosome secretion could attenuate exosomes-mediated radioresistance in the recipient cells. As expected, GW4869, an exosome releasing inhibitor, reduced the radioresistance of bystander cells induced by a hypoxic conditioned medium (Supplementary Fig. s6). Moreover, The expressions of ANGPTL4 and ferroptosis inhibition marker proteins GPX4, SLC7A11, FTH1 and FTL were also increased in these bystander cells that received both N_2_-EXO and O_2_-EXO, while the induction of N_2_-EXO were more pronounced (Fig. 5c and Supplementary Fig. s7). These results indicated that hypoxic exosomes were much more effective in protecting these bystander cells from radiation damage than normoxic exosomes.

Based on our aforementioned data, we speculate that the exosomes carrying ANGPTL4 protein are responsible for the increases of ANGPTL4 in the bystander cells. In support of this assumption, we detected the transcription and protein levels of ANGPTL4 after co-culturing A549 cells with its exogenous N_2_-EXO at different times. After 12 h of co-culture, both expression levels of the protein and mRNA of ANGPTL4 were enhanced. Notably, the total amount of ANGPTL4 protein was significantly increased prior to the increase of corresponding mRNA (Fig. 5d), suggesting that a part of ANGPTL4 protein in the bystander cells might come from the exogenous N_2_-EXO and the others might be generated from the bystander cells as a stress response to exosomes.

To confirm the role of exosomal ANGPTL4 protein in the bystander responses, we extracted the exosomes from A549 and H1299 cells stably transfected with ANGPTL4-OE or sh-ANGPTL4 lentivirus and identified that the content of ANGPTL4 protein in exosomes was increased by ANGPTL4 upregulation under normoxic condition but reduced by ANGPTL4 knockdown under hypoxic condition (Fig. 5e). Again, we cultured normoxic A549 cells with same amount of exosomes but containing different amount of ANGPTL4 for 24 h and then irradiated these recipient cells with 6 Gy γ-rays. It was found that the survival of irradiated cells was enhanced by the normoxic exosomes with ANGPTL4 overexpression (O_2_-A4-OE-EXO) but was attenuated by the hypoxic exosomes with ANGPTL4 knockdown (N_2_-sh-A4-EXO) (Fig. 5f).

In addition, irradiation inhibited the expression of GPX4 and activated the ferroptosis pathway in the bystander A549 cells (Fig. 5g). After treatment with N_2_-EXO derived from A549 cells, the level of GPX4 in the bystander cells was increased, indicating ferroptosis inhibition. However, when the content of ANGPLTL4 in N_2_-EXO was downregulated, the above effects of N_2_-EXO on the increase of GPX4 and inhibition of ferroptosis were reversed. Similarly, the increase of ANGPTL4 content in O_2_-EXO significantly enhanced the expression of GPX4 in the bystander cells and inhibited the occurrence of ferroptosis. As a positive control, the treatment of A549 cells with human recombinant ANGPTL4 protein (rANGPTL4) significantly increased the cellular levels of GPX4 and FTH1 and thus weakened ferroptosis induction. Moreover, silencing GPX4 in the bystander cells significantly attenuated the radioprotection function of exogenous N_2_-EXO (Fig. 5h). Taken together, the radioresistance in NSCLC cells could be elevated by the exosomes released from surrounding hypoxic cells in an ANGPTL4-GPX4-dependent manner.

### Exosomal ANGPTL4 contributed to radioresistance via inhibition of ferroptosis in vivo

**Fig. 6:**
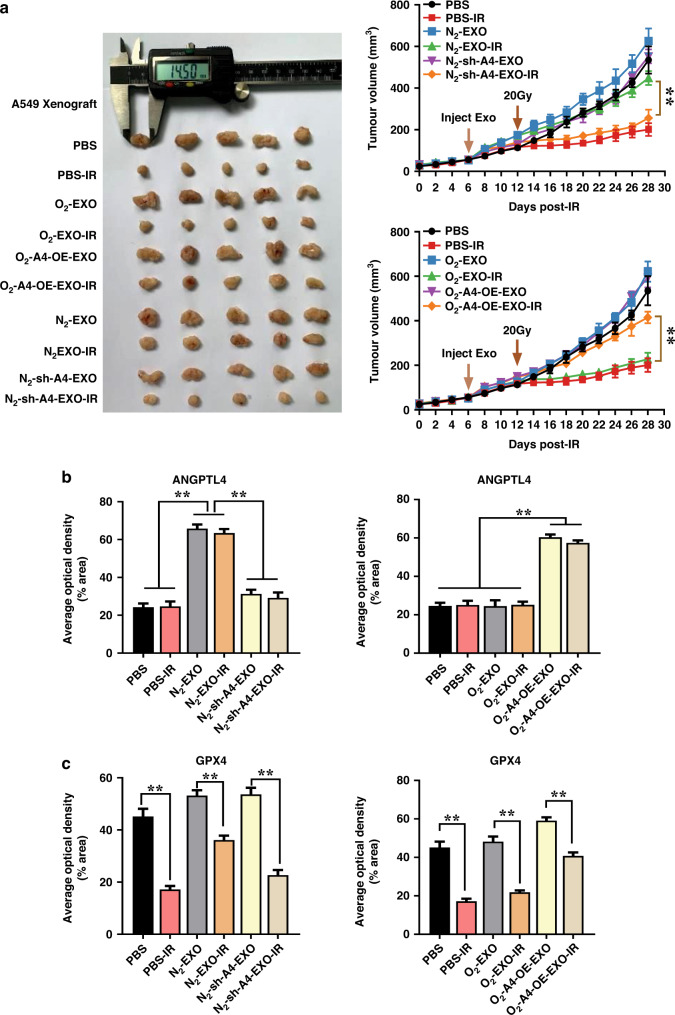
Exosomal ANGPTL4 contributes to radioresistance via inhibition of ferroptosis in vivo. **a** Representative xenograft tumour mass (*n* = 5) and the growth curves of A549 tumours injected with PBS or homologous exosomes (10 μg) once daily for 3 days before local irradiation (20 Gy) followed by continued daily injection until mice sacrifice. Exosomes were generated from normoxic A549 cells with ANGPTL4 overexpression and hypoxic A549 cells with ANGPTL4 knockdown, respectively. **b**, **c** Quantification of immunohistochemical staining of GPX4 and ANGPTL4 in A549 xenograft tumours injected with PBS or indicated exosomes as above. **P < 0.01.

To confirm whether the exogenous exosomal ANGPTL4 could increase the radioresistance of A549 xenograft tumours in mice, we injected different homologous exosomes into the tumour once daily for 3 days before irradiation followed by continually daily injection until mice sacrifice. It was found that the injection of N_2_-EXO but not O_2_-EXO or PBS drastically enhanced the radioresistance of A549 xenograft tumours, while N_2_-sh-A4-EXO treatment had no influence on the growth of irradiated tumour. On the contrary, injection of O_2_-A4-OE-EXO restored the growth of irradiated tumour compared to O_2_-EXO treatment (Fig. 6a). TGDs of PBS-IR, O_2_-EXO-IR, O_2_-A4-OE-EXO-IR, N_2_-EXO-IR, and N_2_-sh-A4-EXO-IR group were 11.08 d, 10.92 d, 4.32 d, 3.88 d and 8.06 d, respectively. Further immunohistochemistry analysis of tumour tissues in the above ten groups revealed that the expressions of ANGPTL4 and GPX4 were significantly increased in the N_2_-EXO and O_2_-A4-OE-EXO groups (Fig. 6b and Supplementary Fig. s8). Collectively, our in vivo results further verified that ANGPTL4 was essential for hypoxic exosomes induced radioresistance in tumours at least partly through GPX4-mediated ferroptosis inhibition.

## Discussion

Globally, lung cancer is still lethal, partially owing to its insidious onset and poor prognosis with a 5-year survival rate of only 4–17%. For most patients, radiotherapy is still considered to be an optimised treatment modality because its indications exist in all stages of lung cancer [29]. However, radioresistance greatly limits the efficacy of radiotherapy in patients with lung cancer. Therefore, revealing the specific mechanism of tumour radioresistance has important clinical implications for formulating and developing new radiotherapy strategies. Hypoxia is a critical cause of radioresistance in solid tumours. Mounting evidence has demonstrated that hypoxia could not only affect the intracellular biological processes directly but also induce tumour radioresistance by remoulding TME [30, 31]. Although hypoxic cells themselves have been extensively studied, the mechanism underlying the distal transfer of radioresistance mediated by the crosstalk between hypoxic cells and normoxic cells under TME remains elusive.

It is well known that ANGPTL4 plays a pivotal carcinogen mediator in tumour growth, proliferation, invasion, angiogenesis and metastasis [32–34]. HIF-1α could directly upregulate ANGPTL4 and promoted tumour metastasis by activating the vascular cell adhesion molecule-1/integrin β1 signalling axis [35]. Expression of ANGPTL4 in breast cancer was enhanced by lncRNA RAB11B-AS1 through increasing recruitment of RNA polymerase II in response to hypoxia, then promoted cancer invasion and distant metastasis [36]. Apart from influencing tumour progression, ANGPTL4 could also serve as predictor of cancer development. Emerging evidence supported that patients harbouring a higher level of ANGPTL4 in various types of tumour were associated with poor prognosis after radiotherapy [37], and ANGPTL4 was expected to be an attractive prognostic or predictive biomarkers and a novel target for cancer treatment. However, studies on the role of ANGPTL4 in tumour radioresistance are hitherto lacking. In this study, both in vivo and in vitro experiments demonstrated that hypoxic TME augmented the intracellular expression of ANGPTL4 (Fig. 1c–f). Given that ANGPTL4 was expressed at a low background level under normoxic condition, we transfected NSCLC cells with ANGPTL4-OE lentivirus under normoxia and sh-ANGPTL4 lentivirus under hypoxia to investigate the biological functions of hypoxia-induced-ANGPTL4 in a positive or negative manner. Specifically, transcriptional inhibition of ANGPTL4 using shRNA under hypoxic condition significantly reversed radioresistance. Conversely, ANGPTL4 overexpression enhanced radioresistance to a level similar to that caused by hypoxic treatment even in normoxic cells (Fig. 2), revealing the essential role of ANGPTL4 in modulation hypoxia-induced radioresistance. It is worthy emphasising that our study proved for the first time that ANGPTL4 could drive NSCLC cells to radioresistance through at least two parallel pathways.

Studies have shown that cell death can be mediated by many ways, such as apoptosis, autophagy, necroptosis and pyroptosis. Recently, the roles of ferroptosis in hypoxia- and radiation-induced cell death have attracted more and more attentions, which contribute to numerous aspects of tumorigenesis, immunity, senescence, and a variety of pathological scenarios [38]. Even so, whether ferroptosis participates in hypoxia-induced radioresistance modulation remains unclear. Growing evidence have discovered that ferroptosis is identified as a natural suppression manner for irradiated solid tumours. Ionising radiation triggers ferroptosis and induces tumour suppression with a better response, and extends patients’ survival after radiotherapy. In recent years, hypoxia has been proved to be one of the factors involved in inhibiting ferroptosis pathway [39, 40]. Our study established a strong link between ferroptosis and hypoxia-induced radioresistance, which was derived from the findings that a robust induction of ferroptosis inhibition-related proteins of GPX4, SLC7A11, FTH1 and FTL under hypoxic NSCLC cells (Fig. 3a). Further lipid peroxidation and cell survival assays demonstrated that ferroptosis inhibition is an important reason of hypoxia-induced radioresistance (Fig. 3b, c). Supportively, we also provided an A549 sh-GPX4 xenograft tumour model to consolidate a negative correlation between ferroptosis and radioresistance (Fig. 3f, g). It was noted that the expression of GPX4 was decreased in the 20-Gy irradiated xenografts but not in the 6 Gy irradiated cells (Fig. 3a, g). This may due to the difference of irradiation dose besides the complex microenvironment of tumour in vivo. Indeed, when the cells were irradiated with a high dose of 12 Gy, the expression of GPX4 was decreased (Supplementary Fig. s9). In addition, we proposed a novel mechanism by which ANGPTL4 contributed to hypoxia-induced radioresistance due to ANGPTL4-elicited ferroptosis inhibition both in vivo and in vitro (Fig. 4). Therefore, upregulation of intracellular ANGPTL4 under hypoxia protected cells from ferroptosis and promoted cell survival during radiotherapy.

When the autocrine regulatory effect of ANGPTL4 becomes explicit, its activity as a paracrine factor in the tumour hypoxic microenvironment is also our main concern. Exosomes can carry a large number of biomolecules, including RNA (miRNA, RNA and lncRNA), proteins and lipids. Due to the protective lipid bilayer membrane, exosomes can stably exist in the peripheral environment of tumours and play an important role in local and remote communication within TME [41]. The yield of tumour-derived exosomes and their protein profiles could be substantially regulated by hypoxia, which further induced biological changes in the hypoxic recipient cells engulfing these exosomes and triggered the bystander effect despite of the barrier of intratumoral heterogeneity of oxygen. Studies have demonstrated that the cargoes of exosomes from hypoxic tumours could be horizontally transferred to normoxic cells, which could activate the malignant transformation of normoxic cells by promoting metastasis [42], cell proliferation [43], and cell viability [44]. Therefore, the transmitted hypoxia-exosomes as mediators in modifying the chemoresistance and radioresistance of normoxic bystander cells are gradually being uncovered [7, 45]. However, the mechanism of radioresistance of NSCLC mediated by hypoxic tumour cell-derived exosomal ANGPTL4 had not been studied yet. Our study revealed that ANGPTL4, as a secretory protein, was not only highly up-expressed in hypoxic lung cancer cells, but also enriched in hypoxic exosomes secreted in the extracellular environment (Fig. 1g). Moreover, the hypoxic exosomal ANGPTL4 protein could be transported to bystander normoxic tumour cells, leading to radioresistance of the neighbours, while this effect was abolished when ANGPTL4 was knocked down in exosomes (Fig. 5b, f). By contrast, after co-culturing with normoxic exosomes with ANGPTL4 overexpression (O_2_-A4-OE-EXO), these recipients exhibited de novo radioresistance, much similar to the radioresistance induced by hypoxic exosomes (Fig. 5b, f). To confirm this effect in vivo, we constructed a xenograft mouse model and obtained results consistent with those in vitro experiments, i.e., the hypoxic exosomal ANGPTL4 drastically enhanced the radioresistance of NSCLC xenograft tumours to irradiation (Fig. 6). Accordingly, our results indicated that ANGPTL4 was crucial for shifting hypoxia-exosomes-induced radioresistance to bystander NSCLC, illustrating a considerable therapeutic window.

Although we have demonstrated that ANGPTL4, as an autocrine factor, contributed to hypoxia-induced radioresistance in lung cancer cells by inhibiting the ferroptosis pathway, the mechanism of ANGPTL4 as a paracrine factor-mediated radioresistance of normoxic lung cancer cells remains unclear. Based on the direct effect of ANGPTL4, we detected various indicators of ferroptosis in the bystander cells. It was noteworthy that hypoxic exosomes could upregulate the intracellular expression of ANGPTL4 in normoxic bystander cells by both directly transferring exosome-ANGPTL4 protein (in a short time) and inducing endogenous synthesis of ANGPTL4 (in a long period) (Fig. 5c, d). Subsequently, GPX4 protein expression in the normoxic bystander cells was further enhanced to inhibit the ferroptosis pathway, resulting in radioresistance of the normoxic bystander cells (Fig. 5c). Of note, hypoxic exosomal ANGPTL4 induced upregulation of GPX4 in the recipients was attenuated when ANGPTL4 in hypoxic exosomes was knocked down (Fig. 5g). Further in vivo experiments confirmed the roles of exosomal ANGPTL4 in the induction of radioresistance and ferroptosis inhibition in NSCLC xenograft tumours (Fig. 6). Combined with the direct function of intracellular ANGPTL4, these results disclosed another parallel pathway of hypoxia-ANGPTL4-mediated radioresistance in NSCLC, suggesting that ANGPTL4 could be transported to bystander normoxic NSCLC through exosomes and subsequently inhibited the occurrence of ferroptosis and promoted the radioresistance of bystander cells. Therefore, the exosomal ANGPTL4 has a potentiality to be a biomarker for predicting the radiotherapy efficacy of solid tumours.

**Fig. 7:**
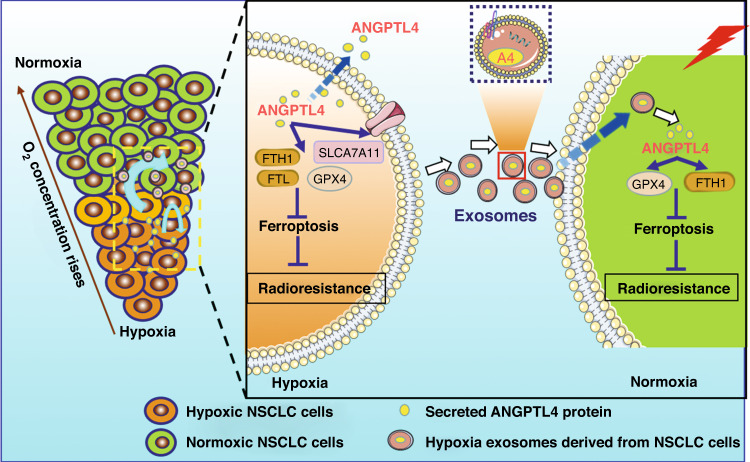
Schematic illustration of the mechanism of ANGPTL4 contributing to radioresistance by inhibiting ferroptosis in hypoxic NSCLC. Solid tumours often contain hypoxic regions and normoxic regions according to the oxygen concentration. Under hypoxia, the expression of ANGPTL4 protein is increased to inhibit cellular ferroptosis, leading to radioresistance of NSCLC. Since ANGPTL4 is a secretory protein, it can be highly contained in the exosomes derived from hypoxic NSCLC. Once the exosomal ANGPTL4 protein is transferred into bystander normoxic tumour cells, it can suppress ferroptosis and thus induce radioresistance of the recipients.

In summary, we disclosed the molecular functions of ANGPTL4 in the hypoxic TME and proposed that hypoxia-induced ANGPTL4 rendered radioresistance of NSCLC through at least two parallel pathways (Fig. 7). First, hypoxia-induced intracellular ANGPTL4-conferred radioresistance by inhibiting ferroptosis. Secondary and more importantly, the exosomes derived from hypoxic NSCLC cells could diametrically transmit radioresistance to surrounding normoxic NSCLC cells through an exosomal ANGPTL4-GPX4-dependent manner. These innovative findings on the relationships among ANGPTL4, ferroptosis, and hypoxia-induced radioresistance have unmasked the roles of intracellular ANGPTL4 and hypoxic exosomal ANGPTL4 in driving resistance to radiotherapy and revealed the potential of exploiting this intrinsic association to improve the clinical outcomes of NSCLC treatment.

## Supplementary information

Supplementary Methods and Figures

checklist

## Data Availability

The data that support the findings of this study are available upon request from the corresponding author. The data are not publicly available due to privacy and ethical restrictions.
