# Supplementary material for: Exosomal protein angiopoietin-like 4 mediated radioresistance of lung cancer by inhibiting ferroptosis under hypoxic microenvironment
Source: Br J Cancer. 2022 Sep 1;127(10):1760–72. doi: 10.1038/s41416-022-01956-7 (PMC9643351; doi:10.1038/s41416-022-01956-7)
Supplement: Supplementary file 1 — Supplementary Methods and Figures [file 41416_2022_1956_MOESM1_ESM.docx]

**Exosomal protein angiopoietin-like 4 mediated radioresistance of lung cancer by inhibiting ferroptosis under hypoxic microenvironment**

Yuhong Zhang, Xinglong Liu, Liang Zeng, Xinrui Zhao, Qianping Chen, Yan Pan, Yang Bai, Chunlin Shao*, Jianghong Zhang*

Institute of Radiation Medicine, Shanghai Medical College, Fudan University, Shanghai 200032, China.

* **Correspondence to:** clshao@shmu.edu.cn (CS); zjh551268@fudan.edu.cn (JZ)

**Supplementary**

1. **MATERIALS AND METHODS**

**Cell culture and irradiation**

Human NSCLC of A549 and H1299 cells were purchased from Cell Bank of Chinese Academy of Science (Shanghai, China) and cultured in RPMI-1640 medium (Gibco, Thermo Fisher Scientific, Waltham, MA, USA) with 10 % fetal bovine serum and 1% penicillin/streptomycin (Gibco) at 37℃ with 5% CO_2_ in a humidified incubator. All human NSCLC cell lines A549 and H1299 were authenticated using Short tandem repeats (STR) profiling in 2019 (Chinese Academy of Science, Shanghai, China). Cells were irradiated with 5 Gy γ-rays (Gammacellt-40; Nordione International Inc., Ottawa, Canada) at a dose rate of 0.70 Gy/min at room temperature**.**

**Hypoxia and drug treatment**

A549 and H1299 cells were maintained in a hypoxia workstation (H35 HEPA hypoxystation, Don Whitley Scientific, Bingley, UK) with a humidified continuous mixture flow of 1% O_2_, 5% CO_2_, and 94% N_2_ at 37°C for 24 h or 48 h based on different purposes. The hypoxic cells were placed in a sealed box for irradiation and then sent back to hypoxia workstation for subsequent treatments.

To explore the role of ferroptosis in radiosensitivity of cells under hypoxic and normoxic conditions, 5 μM RSL3 (ferroptosis activator; Selleck, Shanghai, China) or 5 μM ferrostatin-1 (Ferr-1) (ferroptosis inhibitor; Abclonal, Wuhan, China) were administrated to the cells for 24 h before irradiation, respectively. The pharmaceutical solvent DMSO (0.1%) was used as a control of RSL3 and Ferr-1.

**Quantitative real-time PCR Assay**

Total cellular RNA was extracted from A549 and H1299 cells using TRIzol reagent (Invitrogen, SanDiego, CA, USA) and reversely transcribed into cDNA with PrimeScipt RT reagent Kit with gDNA Eraser (Takara, Otsu, Japan) according to the manufacturer’s instruction. The following primers were used in this study. For the *ANGPTL4* gene, the forward primer was 5’-GCC UGC AGA CAC AAC UCA ATT-3’ and the reverse primer was 5’-UUG AGU UGU GUC UGC AGG CTT-3’. For *β-actin* gene, the forward primer was 5’-CAT GTA CGT TGC TAT CCA GGC-3’ and the reverse primer was 5’- CTC CTT AAT GTC ACG CAC GAT-3’. For *GPX4* gene, the forward primer was 5’-GCC TTC CCG TGT AAC CAG T-3’ and the reverse primer was 5’- GCG AAC TCT TTG AIC TCT TCG T-3’.

**Western blot analysis**

Total cellular proteins were extracted with RIPA buffer containing 100 mM phenylmethanesulfonyl fluoride (PMSF) (Beyotime Biotechnology, Haimen, China) according to the manufacturer’s instruction. An equal amount of protein samples was subjected to 10% SDS-PAGE gel and subsequently transferred to polyvinylidene difluoride (PVDF) membrane (Immobilon-P; Millipore Corporation). After blocked with 5% skimmed milk for 2 h, the membranes were incubated with primary antibodies against GPX4, FTH1, FTL (1:1000, all from Abclonal Technology, Wuhan, China), ANGPTL4 and SLC7A11 (1:1000, both from Proteintech, Shanghai, China), CD63, CD9 (1:1000, both from Cell signaling Technology, Inc., Danvers, MA, USA) and Tubulin (1:1000, Beyotime Biotechnology) at 4℃ overnight. Following incubation with horseradish peroxidase-conjugated secondary antibodies (1:3000, Beyotime Biotechnology) for 1.5 h at room temperature, the proteins were detected using an ECL kit (Millipore, St. Louis, MO, United States) and the black band were analyzed with the Bio-Rad ChemiDoc XRS system.

**Extraction and identification of exosomes**

The supernatants of cells with different treatments were collected in 50 ml tubes, centrifuged at 300 g for 10 min, 2000 g for 20 min, and 12000 g for 1 h. Then the supernatants were collected and concentrated at 3000 g for 15 min using an Amicon® Ultra-15 Centrifugal Filter (Millipore, Massachusetts, America) followed by the isolation using an Exosome Isolation Kit (Umibo, Shanghai, China) according to the manufacturer’s instruction. The exosomal protein concentration was measured using a BCA Protein Quantitation Kit (Thermo Fisher Scientific Inc., Waltham, MA) according to the manufacturer’s instruction. The biomarker proteins of exosomes were identified by Western blotting assay.

**Tandem Mass Tag (TMT) Quantitative Proteomic Analysis**

Protein mass spectrometry was performed by a qualified company (PTM Biolabs, Hangzhou, China). Briefly, exosomes were extracted from normoxic and hypoxic A549 cells and the the exosomal protein concentration was measured using a BCA Protein Quantitation Kit (Thermo Fisher Scientific Inc., Waltham, MA) according to the manufacturer’s instruction. Then the equal quantity of exosomes samples were subjected to trypsin digestion, TMT tags labeling, LC-MS/MS analysis by a high-resolution mass spectrometer Q Exactive plus (Thermo Fisher Scientific) and then subsequent database comparison.

**Exosomes uptake**

The purified exosomes were labeled with PKH26 (Sigma, USA). Briefly, Exosomes were mixed with PKH26 for 10 min in dark and then resuspended by PBS. The excess PKH26 dye was removed by ultracentrifugation at 35000 rpm for 4 h at 4℃ (Beckman OptimaXE-100/XE-90, USA). The exosome pellets were washed triply with PBS. H1299 cells were co-cultured with PKH26-labeled-exosomes for 2 h or 6 h and then fixed with 4% paraformaldehyde. Cell nuclei were labeled with DAPI dihydrochloride (Beyotime Biotechnology). Exosomes uptake was visualized and photographed using a high-content imaging system (ImageXpress Micro 4, Molecular Devices, San Francisco, CA, USA).

**ELISA assay of ANGPTL4**

Exosomes and cell supernatants were obtained from cell culture medium under normoxic and hypoxic conditions for 48 h. The concentrations of ANGPTL4 in exosomes and cell supernatants were measured by a Human ANGPTL4 ELISA kit (Proteintech, Rosemont, United States) according to the manufacturer’s instruction. This kit has a detection sensitivity from 0.313 ng/mL to 20 ng/ml of ANGPTL4. The optical density (OD) of sample was measured at 450 nm using the microplate reader.

**Immunofluorescence (IF) assay**

NSCLC cells were seeded in 24-well plates overnight prior to normoxic or hypoxic treatment for 24 h. Cells were washed with precooled PBS for 10 min and fixed with 4% paraformaldehyde for 15 min at room temperature, then permeabilized with 0.5% Triton X-100/PBS for 10 min. After permeabilization, the cells were treated with blocking buffer for 30 min and incubated with ANGPTL4 rabbit antibodies (1:1000, Proteintech) at 4 ℃ overnight. Then fluorescent secondary antibody was added to the 24-well plate to bind the primary antibody in dark for 1 h at room temperature. The nuclei were labeled with DAPI dihydrochloride (Beyotime Biotechnology). Cell fluorescence image was observed randomly with a high-content imaging system (ImageXpress Micro 4).

For IF assay of tumor tissue, mice xenograft tumor samples were fixed in formalin and embedded in paraffin followed by cutting into 4-μm thick tissue slices. The tissue sections were incubated with primary antibodies against HIF-1α (1:100, Abcam, Cambridge, MA, USA) and ANGPTL4 (1:100, Proteintech) at 4℃ overnight. Then tissue sections were washed triply with PBST and incubated with HRP-labeled secondary goat antibody (1:1000, Jackson ImmunoResearch Laboratories, Inc. Shanghai, China) for 50 min at room temperature, followed by the treatment with DAPI (Beyotime Biotechnology) for 10 min. Tissue images in at least three fields were randomly captured at 400X magnification using a fluorescence microscope (Nikon Eclipse CI-S, Japan) and analyzed with the ImageJ software.

**Construction of gene overexpression or knockdown cell lines**

The lentivirus containing ANGPTL4 interference (sh-A4), ANGPTL4 overexpression (A4-OE), or GPX4 interference (sh-GPX4) were purchased from Hanbio Biotechnology Co., LTD (Shanghai, China). Their negative control had random sequences. Briefly, A549 or H1299 cells were infected with the lentivirus for 24 h according to the manufacturer's instruction. When these A549 or H1299 cells could stably grow in medium containing 3 µg/ml blasticidin and puromycin to exclude any off-targeted cells, the culture medium was replaced with RPMI-1640 for cell culture of another week. Western blot and qRT-PCR assays were carried out to identify the efficiency of knockdown or overexpression of the targeting gene.

**Animal experiments**

Five-week-old athymic male nude mice (BALB/C-nu/nu) (SIPPR/BK Lab. Animal Co. Ltd., Shanghai, China) were maintained at a standard condition (24˚C temperature, 50% relative humidity and 12 h light/dark cycle) for one week before experiments. For conducting the xenograft model, A549 cells (6×10^6^/100 μl) with or without ANGPTL4 overexpression (A549-A4-OE) and GPX4 interference (A549-sh-GPX4) were subcutaneously injected into the left flank of nude mice. When the tumor size reached about 100 mm^3^, the nude mice were randomly divided into two groups: Non-IR group (n=5) and IR group (n=5). The mice in IR group were anesthetized with ketamine/xylazine (100 mg/kg + 10 mg/kg) and placed in well-ventilated lead mould, allowing local irradiation of xenograft with a single dose of 20 Gy X-rays (X-RAD 320, Precision X-Ray, Inc., North Branford, CT, USA; 12 mA, 2-mm aluminum filtration) at a dose rate of 0.883 Gy/min.

To know the effect of exosomal ANGPTL4 on tumor radioresistance, exosomes were extracted from different treatment groups including O_2_-EXO, O_2_-A4-OE-EXO, N_2_-EXO and N_2_-sh-A4-EXO. Exosomes of O_2_-EXO and O_2_-A4-OE-EXO were extracted from A549 cells stably transfected with ANGPTL4-OE or NC under normoxia. Exosomes of N_2_-sh-A4-EXO and N_2_-EXO groups were extracted from A549 cells stably transfected with sh-A4 or shNC under hypoxia. The mice were randomly divided into 10 groups (n = 5/group) with the following treatments: PBS control (PBS), PBS and irradiation (PBS-IR), normoxic exosomes (O_2_-EXO), normoxic exosomes and irradiation (O_2_-EXO-IR), normoxic exosomes with ANGPTL4 overexpression (O_2_-A4-OE-EXO), normoxic exosomes with ANGPTL4 overexpression and irradiation (O_2_-A4-OE-EXO-IR), hypoxic exosomes (N_2_-EXO), hypoxic exosomes and irradiation (N_2_-EXO-IR), hypoxic exosomes with ANGPTL4 knockdown (N_2_-sh-A4-EXO), and hypoxic exosomes with ANGPTL4 knockdown and irradiation (N_2_-sh-A4-EXO-IR). When tumor size reached about 50 mm^3^, 20 μl of exosomes (10 μg) from different groups or 20 μl of PBS was injected into the tumor once daily for 3 days before irradiation followed by continuous daily injection until the endpoint as indicated. The tumor volumes were measured every other day and calculated with the equation: V=(L×W^2^) × π/6 (L= length, W= width). Tumor growth delayed (TGD) for each treatment was calculated as TGD = [T_tv_ ×5]-[T_cv_ ×5], where T_tv_×5 and T_cv_×5 is the time to reach five-fold tumor volume increase from starting time point based on an exponential growth fit in the treated tumors (tv) and untreated control tumors (cv), respectively. When T_tv_×5 was not reached, the volume at the ending time of experiment was used. The mice were sacrificed by cervical dislocation when tumor volume approached to about 1000 mm^3^. Then, the xenograft tumor was dissected, photographed and subjected to immunohistochemical analysis.

Animal experimental procedures and operations without blinding were approved by the Animal Welfare and Ethics Committee of Fudan University, complied with the ARRIVE guidelines, and carried out in accordance with the National Institutes of Health guide for the care and use of Laboratory animals.

**Colony formation assay**

The radiosensitivity of A549 and H1299 cells were determined using a colony formation assay. For the effect of ANGPTL4 on radioresistance, cells stably transfected with A4-OE or sh-A4 were incubated under normoxic and hypoxic conditions for 24 h, then irradiated with 2, 4, 6, 8 Gy of γ-rays, followed by trypsin to generate a single cell suspension and seeded in 6-well plates in triplicate. For the effect of ferroptosis on radioresistance under hypoxic condition, cells were seeded in 60-mm culture dish overnight and then pretreated with ferroptosis inducer RSL3 or ferroptosis inhibitor Ferr-1 for 24 h prior to 6 Gy γ-ray irradiation under normoxic or hypoxic condition, then reseeded and cultured at 37 °C for 2 weeks, fixed with methanol for 20 min, and stained with 0.5% crystal violet for 30 min. Cell colonies (Count > 50 cells) in each well were counted visually. The surviving fraction was calculated with the single-hit multitarget model SF=1-(1-exp(-k*D))^N^ and normalized to that of unirradiated control cells.

**Cell proliferation assay**

1500 cells per well in six replicates were seeded in 96-well plates and incubated at 37°C for 72 h, and then the cell activity was measured using a Cell Counting Assay Kit-8 (CCK-8; Yesen, Shanghai, China) according to the manufacturer’s instruction. The optical density (OD) of sample was measured at 450 nm using the microplate reader.

**Generation of conditioned medium**

After hypoxia treatment for 48 h with or without 5 μM GW4869 (Sigma-Aldrich Corp, St. Louis, USA), the conditioned medium was collected from A549 and H1299 cells and filtered through a 0.2-µm filter.

**Immunohistochemistry**

Immunohistochemical staining of four proteins (ANGPTL4, GPX4, FTH1, and Ki67) were performed on the formalin-fixed and paraffin-embedded tissue sections from xenograft tumors according to manufacturer’s instruction. Briefly, the tissue sections were incubated with primary antibodies against ANGPTL4 (1:100), GPX4 (1:100), FTH1 (1:100), Ki67 (1:100) at 4℃ overnight, then washed triply with PBST and incubated with HRP-conjugated anti-rabbit IgG for 50 min at room temperature followed by the incubation of diaminobenzidine working solution (DAKO, Glostrup, Denmark). Finally, the tissue sections were immersed in 3’,3’-diaminobenzidine (Sigma-Aldrich Corp, St. Louis, USA) and counterstained with hematoxylin. Each tumor tissue slide was photographed randomly in at least 5 fields using a microscope (Nikon Eclipse CI-S, Japan) and the relative average optical densities of immunohistochemical staining images were analyzed with ImageJ software.

**Detection of lipid peroxidation**

To study the relevance of hypoxia and ferroptosis, cells were divided into 10 groups based on the indicated treatments (Ctrl-normoxia, IR-normoxia, RSL3-normoxia, Ferr-1-normoxia, IR-normoxia-Ferr-1, Ctrl-hypoxia, IR-hypoxia, RSL3-hypoxia, Ferr-1-hypoxia, and IR-hypoxia-Ferr-1). To study the relationship of ANGPTL4 and ferroptosis, A549/H1299 (A4-OE) and their control cells were divided into 4 groups based on the indicated treatments (NC-normoxia-IR, A4-OE-normoxia-IR, shNC-hypoxia-IR, and sh-A4-hypoxia-IR). A549 and H1299 cells were treated with 5 μM RSL3 or 5 μM Ferr-1 for 24 h prior to 6 Gy γ-ray irradiation under normoxic or hypoxic condition. A549/H1299 (A4-OE) and their control cells were exposed to 6 Gy γ-rays under normoxia or hypoxia, then these cells were washed by PBS and incubated with 5 μM lipid peroxidation probe C11 BODIPY 581/591 (Abclonal technology, Wuhan, China) for 30 min in a humidified incubator at 37 ℃. The cellular level of lipid peroxidation was detected with a high-content imaging system (ImageXpress Micro 4) and evaluated by the ratio of green fluorescence (oxidized C11 BODIPY) to red fluorescence (reduced C11 BODIPY), whose high level indicates an increase in accumulation of lipid peroxidation and the level of ferroptosis.

**STR identification**

All human NSCLC cell lines A549 and H1299 were authenticated using Short tandem repeats (STR) profiling in 2019 (Chinese Academy of Science, Shanghai, China) and the results for A549 and H1299 were as follows: A549, D5S818: 11,11; D13S317: 11,11; D7S820: 8,11; D16S539: 11,12; VWA: 14,14; TH01: 8,9.3; AM: X,Y; TPOX: 8,11; CSF1PO: 10,12; H1299, D5S818: 11,11; D13S317: 12,12; D7S820: 10,10; D16S539: 12,13; VWA: 16,17,18; TH01: 6,9.3; AM: X,X; TPOX: 8,8; CSF1PO: 12,12.

**Statistical analysis**

Results of cell culture experiments were collected from at least 3 independent replicates. Tumor volumes from at least 5 individual mice in each group were plotted. All data met the assumptions of the tests and were presented as mean ± SD. Student’s t-test was used to determine the statistical significance (GraphPad Prism 7.00). *P*＜0.05 was considered as statistically significant.

1. **Supplementary figures**

**
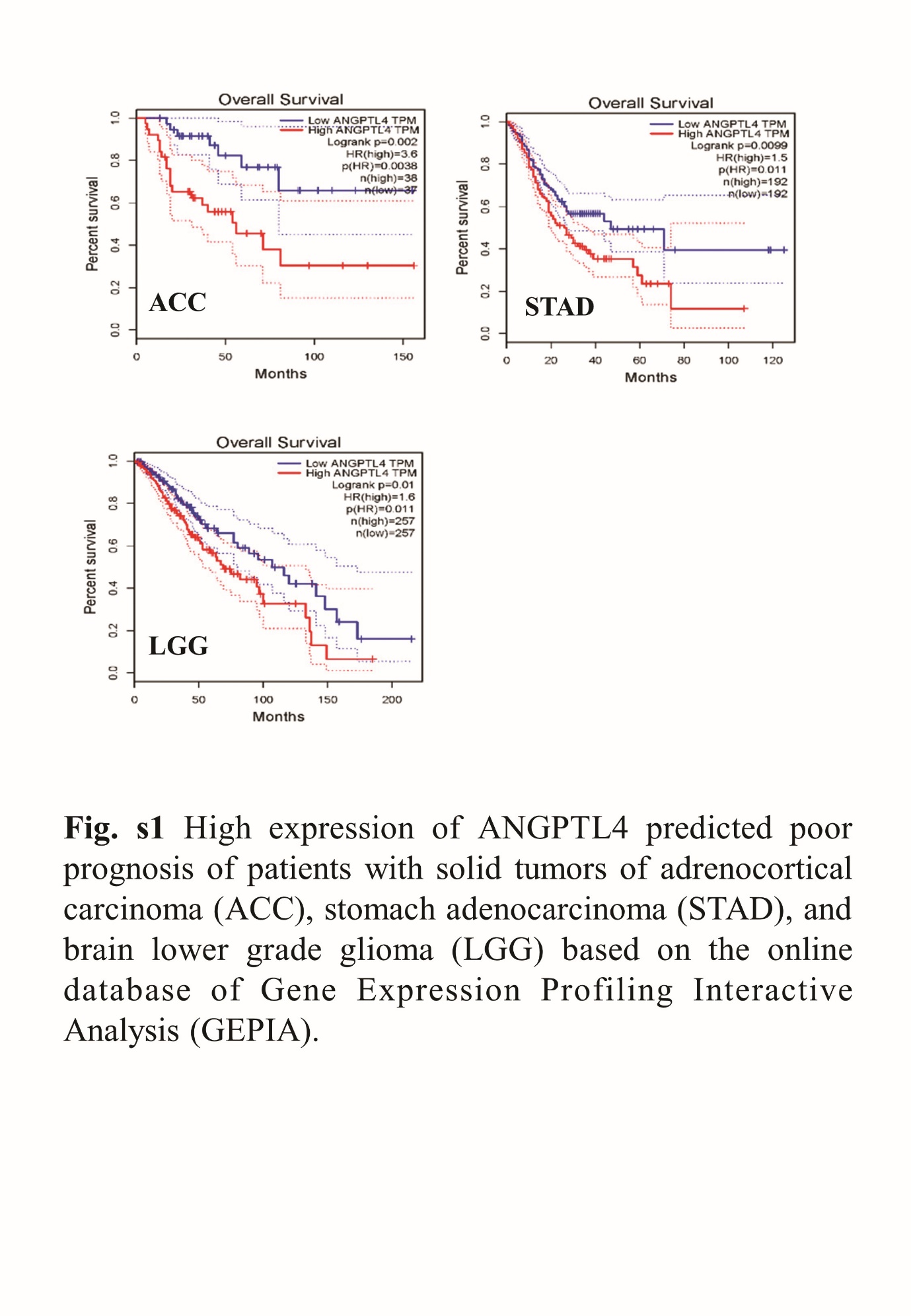
**

**Fig. s1 High expression of ANGPTL4 predicted poor prognosis.** Patients with solid tumors of adrenocortical carcinoma (ACC), stomach adenocarcinoma (STAD), and brain lower grade glioma (LGG) are based on the online database of Gene Expression Profiling Interactive Analysis (GEPIA).


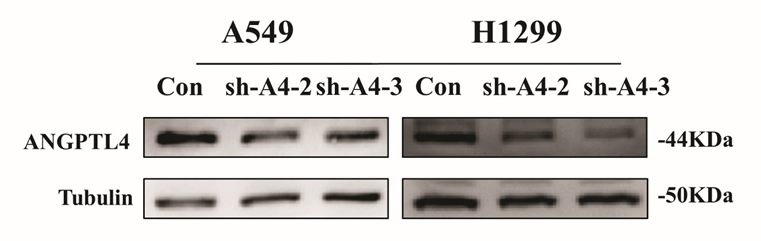


**Fig. s2** **Western blot analysis of ANGPTL4 expression.** A549 and H1299 cells transfected with sh-ANGPTL4-2, sh-ANGPTL4-3 or its control (Con).


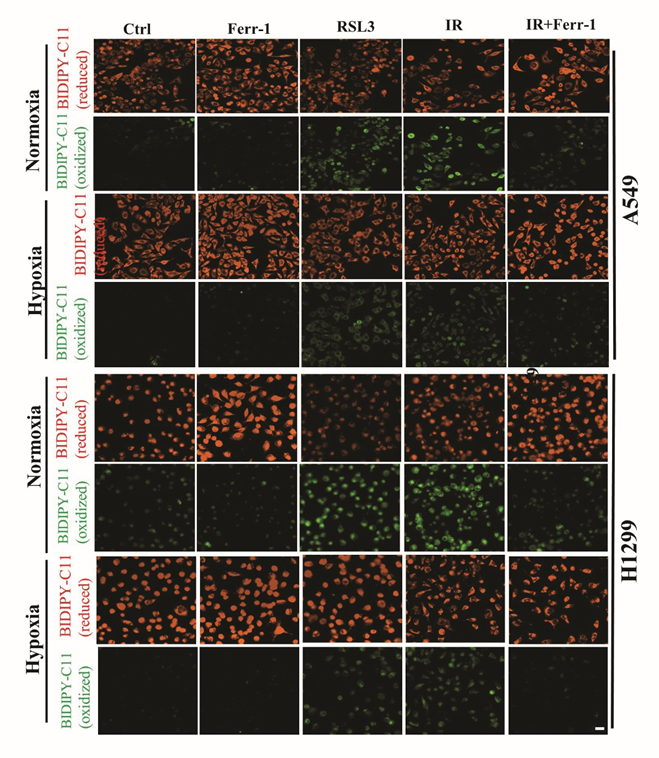


**Fig. s3 Hypoxia promoted radioresistance through inhibition of ferroptosis.** Detection of lipid peroxidation in normoxic and hypoxic A549 and H1299 cells pretreated with ferrostatin-1 and RSL3 for 24 h followed by 6 Gy γ-rays irradiation under.


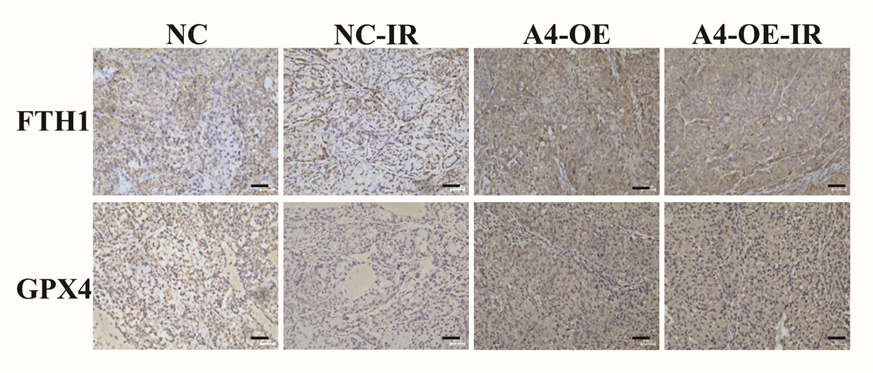


**Fig. s4 ANGPTL4 induced the expression of FTH1 and GPX4 *in vivo*.** Representative images of immunohistochemical staining (FTH1 and GPX4) in xenograft tumors of A549 cells transfected with ANGPTL4-OE or its negative control (NC). Scale bars, 50 μm. Tumors of IR group were locally irradiated with X-rays at a single dose of 20 Gy.


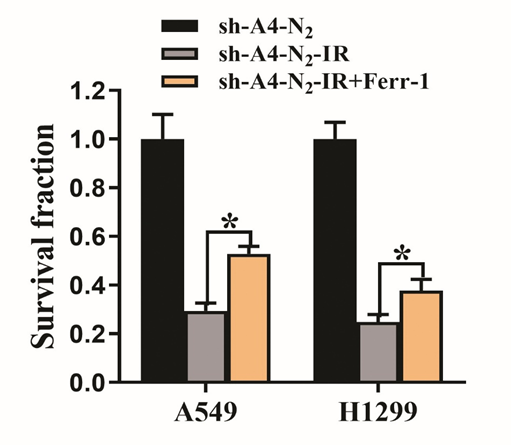


**Fig. s5** **Treatment of sh-ANGPTL4 transfected cells with ferroptosis inhibitor Ferr-1 restored hypoxia induced radioresistance.** Clonogenic survivals of hypoxic A549 and H1299 cells were stably transfected with sh-ANGPTL4 and treated with or without Ferr-1 followed by 6 Gy γ-ray irradiation. * *P* < 0.05.

**
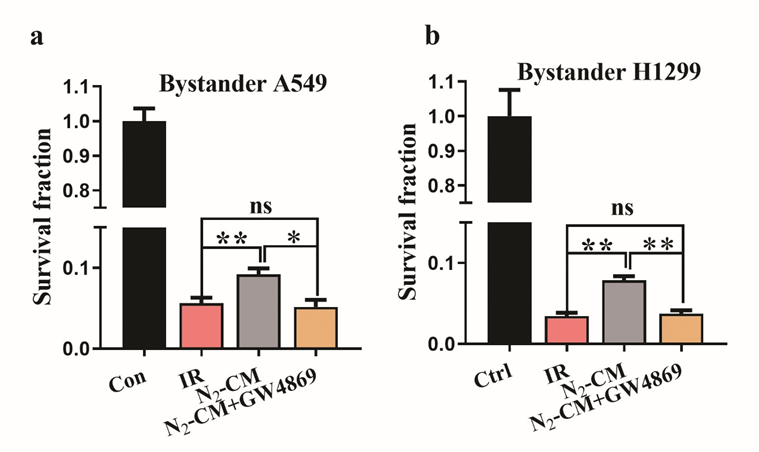
Fig. s6 Hypoxic exosomes was essential for the radioresistance of bystander cells.** Clonogenic survivals of A549 (a) and H1299 (b) cells that were co-cultured with homologous hypoxic conditioned medium (N_2_-CM) or hypoxic conditioned medium with GW4869 (N_2_-CM-GW4869) for 24 h followed by 6 Gy γ-ray irradiation. ** *P* < 0.01.

1. **b**

**
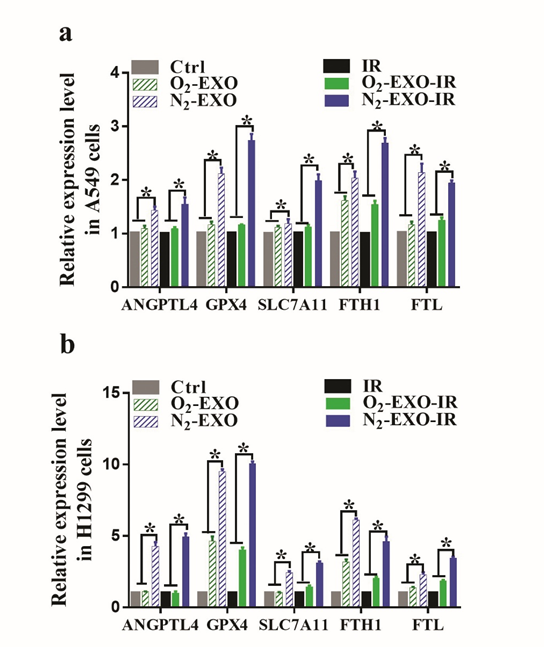
**

**Fig. s7 Relative expression levels of ferroptosis inhibition proteins of GPX4, SLC7A11, FTH1, FTL, and ANGPTL4 protein in NSCLC cells.** A549 (**a**) and H1299 cells (**b**) that were co-cultured with its homologous O_2_-EXO and N_2_-EXO followed by 6 Gy of γ-rays irradiation. * *P* < 0.05.


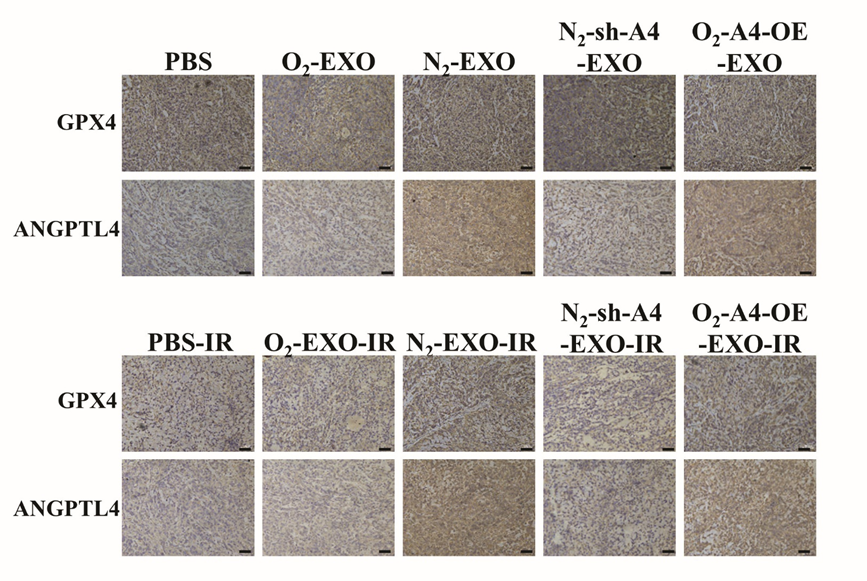
**Fig. s8 Representative immunohistochemical images of GPX4 and ANGPTL4 staining in A549 xenograft tumors**. The tumors were injected with PBS or homologous exosomes (10 μg) once daily for 3 days before local irradiation (20 Gy) followed by continuous daily injection until mice sacrifice. Exosomes were generated from normoxic A549 cells with ANGPTL4 overexpression (A4-OE) and hypoxic A549 cells with ANGPTL4 knockdown (sh-A4), respectively. Scale bars, 50 μm.

**
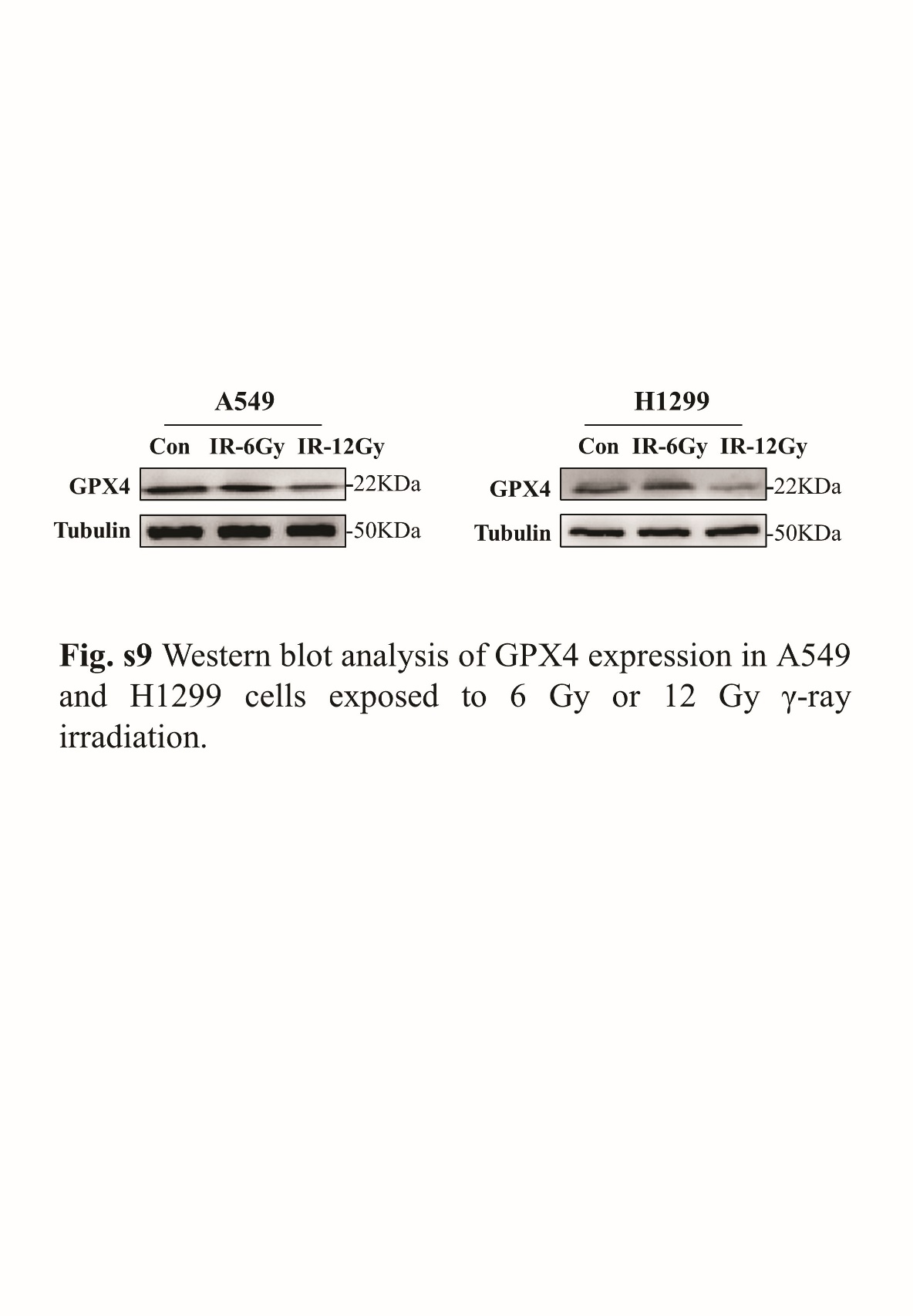
**

**Fig. s9 Western blot analysis of GPX4 expression.** A549 and H1299 cells exposed to 6 Gy or 12 Gy γ-ray irradiation.
